# Government Trust, Environmental Pollution Perception, and Environmental Governance Satisfaction

**DOI:** 10.3390/ijerph19169929

**Published:** 2022-08-11

**Authors:** Haibo Ruan, Li Qiu, Jun Chen, Shuo Liu, Zhiyuan Ma

**Affiliations:** 1 Institute of China Rural Studies, Central China Normal University, Wuhan 430079, China; 2 Institute of School of Education, Central China Normal University, Wuhan 430079, China

**Keywords:** government trust, environmental pollution perception, environmental governance satisfaction

## Abstract

Environmental governance is related to the healthy living standard of human beings and the sustainable development of an economic society. It is of great significance to explore the influence of government trust and environmental pollution perception on environmental governance satisfaction to improve the performance of government environmental governance. Based on the CSS2019 survey data, 3872 survey samples were statistically analyzed, and the optimal scale regression model was used to analyze the relationship between government trust, environmental pollution perception, and environmental governance satisfaction. The results showed that 52.27% of the respondents believed that the satisfaction of environmental governance was good, and both government trust and environmental pollution perception had significant positive effects on the satisfaction of environmental governance. The trust level of the central government, district and county governments, and township governments shows a “differential government trust” state, which is pyramidal. However, the impact of government trust on environmental governance satisfaction shows an inverted pyramid structure, and the township government has the largest effect, which is not matched with the distribution of government trust level. The influence effect of air pollution perception is relatively large, and the public is sensitive to air pollution. Government trust has an impact on the satisfaction of environmental governance through the “expectation-response” path. People are close to the township government and have the opportunity to contact and interact with the township government and its staff. They can directly observe the governance performance and share the public goods of environmental governance. Therefore, it is necessary to further improve the trust level of township governments, strengthen the control of air pollution and improve the township government’s environmental governance ability.

## 1. Introduction

Good health and well-being is the human pursuit of a better life. The environmental problems represented by air pollution have seriously affected the sustainable development of economy and human health [1]. In December 2017, the third United Nations Environment Conference was held in Nairobi, the capital of Kenya. The theme of the conference was “Towards a Zero Pollution Earth”. The General Assembly recognized that human pollution is a serious challenge. The welfare loss caused by environmental pollution is estimated to exceed USD 4.6 trillion annually. What is more serious is that environmental degradation has led to the loss of a large number of people’s lives, and the deterioration of air quality and air pollution from dust storms pose a major health threat. The main pollution sources come from the production, life, and other activities to the environment pollutants [2], including industrial pollution, agricultural pollution, and other emissions of harmful gases, industrial wastewater, solid waste, etc. [3]. According to a 2016 report by the World Health Organization (WHO), 92% of the world’s population lived in environments with excessive air quality, and approximately three million deaths a year were related to exposure to outdoor air pollution, accounting for up to two thirds of global deaths related to air pollution in Southeast Asia and the Western Pacific regions [4]. Therefore, the World Health Organization calls on all governments to act together to combat environmental pollution and reduce the threat of environmental pollution to human living conditions [5].

The problem of environmental pollution must be solved to realize the sustainable development of human beings, and the treatment of environmental pollution has become an important responsibility of the government. For the government, it will reduce government trust, undermine government legitimacy, and increase public dissatisfaction without taking active measures to deal with environmental pollution. Some scholars have found that, on the one hand, the problem of environmental pollution is getting worse; on the other hand, people’s awareness of environmental protection is gradually increasing. When people perceive the severity of environmental pollution, their satisfaction with environmental governance will be seriously weakened and their trust in the government will be further affected [6]. A higher citizen satisfaction will bring greater government trust, which means the improvement of government performance and governance level [7]. Environmental governance is related to people’s evaluation attitude, and environmental governance satisfaction is not only related to potential public opinion, but also an important source of government credibility [8]. Then, in turn, does government trust affect environmental governance satisfaction?

In 2013, the Chinese government formulated the Action Plan for Air Pollution Prevention and Control, and in 2014 revised the Environmental Protection Law. In 2015, the Chinese government put forward the concept of green development, emphasizing the relationship between man and nature, development and the environment, and making development more coordinated and sustainable. In 2018, the Chinese government issued the Three-year Action Plan for Improving Rural Living Environment, which calls for centralized improvement of rural garbage and sewage treatment and village appearance. The government began to invest a lot of manpower, material resources, and financial resources to focus on environmental pollution. On this basis, the government has proposed a five-year environmental improvement campaign to ensure the success of environmental governance. The Chinese government has included green and ecological development indicators in government performance appraisals as an important part of promotion appraisals for local officials. The performance of government in pollution control should not only be assessed by the upper bureaucratic government, but also by the public’s satisfaction with environmental governance [9]. Environmental governance is an important part of the government’s public services, and public satisfaction is an effective evaluation of the government’s pollution control. The improvement of public satisfaction has become an important goal pursued by government departments and an important index to evaluate the performance of government governance [10]. In terms of satisfaction with environmental governance, the academic community mainly discusses the following three aspects:

One is the impact of individual factors on the satisfaction of environmental governance. Ernst found that individual age affected the satisfaction of environmental governance. With the increase in age, the greater the impact of environmental pollution, and the lower the evaluation of the satisfaction of environmental governance [11]. Su et al. found that gender has a significant impact on the satisfaction of environmental governance. Men’s satisfaction is lower, while women’s satisfaction is higher, which is due to the differences in social division of labor and occupation between men and women, or the influence brought by the working environment [12]. The study of Zhan et al. found that there is a positive correlation between people’s education level and their satisfaction with environmental governance, and education level can improve people’s environmental awareness [13]. Geng et al. found that environmental awareness has a negative and significant impact on the satisfaction of environmental governance. People with a stronger environmental awareness have a lower satisfaction with environmental governance, which lies in their higher environmental protection concept [14]. Du further found that people with higher environmental awareness were more aware of the harm of pollution and regarded pollution as a serious threat to their survival and development [15]. Tang et al. found that Internet use has a negative and significant impact on the satisfaction of environmental governance. People can learn about environmental pollution problems in the society through the Internet, increase their negative news, and reduce their favorable feelings toward the government’s environmental governance [16].

Second, the impact of the environmental governance system on the satisfaction of environmental governance. Traditional environmental governance is dominated by the government and has not yet formed a diversified governance system. As Leng said, in the past, environmental governance was promoted from top to bottom by the government, and the public was excluded from environmental governance. Modern environmental governance needs to involve the public in the environmental governance system, which can play the role of pressure transmission and supervision and urge the implementation of environmental protection policies [17]. Yin et al., found that by mobilizing the public to participate in environmental governance through education, publicity, mobilization, and incentives, etc., the government can effectively improve the public’s awareness of environmental protection and improve the performance of environmental governance [18]. Wu believes that the quality and satisfaction of environmental governance can be effectively improved by incorporating the public into the environmental governance system as a party of diversified governance and giving play to the subjective initiative of the public [19]. Gao found that Chinese people’s participation in environmental governance through social and non-governmental organizations can achieve interaction with the government, realize accountability to the government, and further promote the success of policies. The combination of institutionalized public participation and environmental governance is conducive to improving the subjective evaluation of the people [20]. The establishment of a diversified environmental governance system of “government, enterprise and public” can improve the public’s support for environmental policies and regulations. There is a correlation between the diversified environmental governance system and the satisfaction degree of environmental governance [21].

Third, the impact of the implementation of policies and measures on environmental satisfaction. Wang et al. believe that the premise of environmental governance is that the government should adopt environmental legislation, law enforcement, and pay corresponding environmental governance funds, and the active actions of the government can improve the subjective impact of the people [22]. According to Li, the government’s environmental governance policies and related policy implementation tools should be recognized by the public, which is not only conducive to the promotion of environmental governance, but also can improve the performance and satisfaction of environmental governance [23]. Just as Chu found in his research, the degree of perfection of environmental policy itself and the degree of conformity between specific content and objective reality, will affect the subjective feelings of the public. For example, the location of the installation of designated garbage bins and the time for cleaning up garbage will directly affect the public’s experience of environmental products [24]. This requires the effectiveness and pertinence of environmental measures. Xu conducted a study on the clean heating policy launched in North China, and he believes that the policy is highly targeted, which has significantly improved the local air quality and people’s satisfaction with environmental governance [25]. Wang et al. believe that the targeted centralized treatment of household garbage and sewage by the government can significantly improve the satisfaction of the public [26].

Previous studies have revealed the influencing factors of environmental governance satisfaction from different aspects. Individual citizens are mainly micro-level influencing factors, such as individual biological characteristics and individual human resource endowment. Government policies are mainly related to macro-level and middle-level influencing factors, such as policy formulation, policy implementation, choice of policy tools, the pertinence and effectiveness of policy implementation, etc. This provides the premise and foundation for the future research. However, there are also shortcomings in the above studies [27]. On the one hand, there are few answers about the impact of government trust on environmental governance satisfaction, especially the relationship between government trust and environmental governance satisfaction at different levels, while government trust is an important source of modern government legitimacy. On the other hand, the influence of the perception of environmental pollution on the satisfaction of environmental governance is rarely discussed from the perspective of subjective and objective relationships, while the perception of environmental pollution can directly reflect the current situation of the government’s environmental governance [28]. Based on this, this study will explore the impact of government trust and environmental pollution perception on environmental governance satisfaction. This study is of great significance for further promoting environmental governance, enhancing the level of trust in government, and improving public satisfaction with environmental governance.

## 2. Theoretical Backgrounds and Hypothesis Development

In 1989, the World Bank first used the term “governance crisis” to describe the situation in Africa, and governance theory began to be widely used in various fields [29]. The typical representatives of governance theory include Rosinau, Roots, Peters, etc. The core point of governance theory is that the governing body of public affairs should include government, enterprises, non-governmental organizations, and individual citizens, and all the subjects should coordinate and cooperate with each other [30]. Environmental governance means that the subject of environmental governance makes environmental governance decisions according to certain principles and systems, and gives certain governance responsibilities to each subject, so as to achieve the maximization of environmental governance and sustainable development of the environment [31]. However, environmental governance may also fail, or policies may fail, so satisfaction with environmental governance becomes an important part of the evaluation of environmental governance. The so-called satisfaction degree of environmental governance refers to the satisfaction degree of people with the environmental pollution control around their lives, including air pollution control, water pollution control, noise pollution control, catering pollution control, and so on [32]. The government plays a leading role in environmental governance and plays a key role [33]. The level of trust in government, then, is crucial.

### 2.1. Government Trust and Satisfaction with Environmental Governance

Boon et al. viewed government trust from the perspective of psychology and understood government trust as a trustworthy positive expectation state of the public towards the government and its behavior [34]. Based on this, Miller said that government trust should be understood as the public’s evaluation of government public services or government performance [35]. In this analytical framework, government trust follows the path of “expectation-response”, where people expect government performance to meet their predetermined needs. The logic of establishing the relationship between government trust and environmental governance satisfaction is government performance and public expectation [36]. With the expansion of modern government functions, environmental governance has been brought into the public functions of the government and become a public product provided by the government. If people are satisfied with the environmental governance behavior of the government, the trust level of the government will be improved. However, not all government performance can bring government trust. Only government performance that meets people’s needs and interests can generate trust [37]. The government’s performance in environmental governance, such as cleaning up urban garbage, controlling air pollution, purifying urban sewage, and checking unqualified environmental protection behaviors of factories, etc., has objectively changed the living environment of the people, fulfilled the promise of government policies, realized policy expectations, and thus improved people’s recognition of the government and generated government trust [38].

High public trust in the government will force the government to maintain its public image of environmental protection and build government authority. The government will also respond to public expectations, further implement environmental governance behaviors, and improve the quality of environmental public service products [39]. The government will also respond to public expectations, further implement environmental governance, and improve the quality of environmental public service products. People’s trust in the government is also a form of accountability. It requires the government to consider people’s demands when formulating environmental policies, protect people’s rights when performing environmental protection functions, increase people’s sense of gain in environmental governance, and improve their happiness. Government trust can increase the motivation of government to take effective measures and minimize the resistance of policy implementation [40]. The research of Lan Gao et al. shows that people who trust the government are more likely to reach an agreement with the government on policies, which means that the government can promote collective action to a certain extent and provide better and more effective public services [41]. In conclusion, under the analysis framework of “forward to–response”, the higher government trust makes the government’s environmental governance more reliable, so the government can assume public responsibilities and achieve public goals. The government will act in the interest of the public and adopt more environmental protection behaviors that meet the needs and expectations of the public, making the government’s behavior more predictable [42]. Based on this, the following hypothesis is proposed:

**Hypothesis** **1** **(H1):** *There is a positive and significant relationship between government trust and environmental governance satisfaction.*

### 2.2. Perception of Environmental Pollution and Satisfaction with Environmental Governance

Environmental pollution perception has become a hot topic in sociology, psychology, public administration, and other disciplines. Environmental pollution perception is the subjective impression formed by individuals based on the quality of the surrounding environment, which is a process from objective to subjective [43]. The perception of environmental pollution comes from the quality of the surrounding environment, which forms the subjective perception after the attitude evaluation and value judgment of the individual brain [44]. According to the research results of John R. Gold, there is a process of environmental pollution perception. The process of environmental perception is that the subject of perception collects the information of the surrounding environment under the stimulation of the physical environment and processes the information to form a psychological environment in the brain, so as to guide and evaluate other behaviors according to it [45]. On this basis, some scholars put forward the hypothesis of environmental pollution driving, whose core point is that environmental quality affects people’s environmental behavior. When people perceive themselves to be exposed to a polluted environment, their environmental experience will be reduced, and their awareness of environmental protection will be stimulated. When people perceive the quality of the surrounding environment to be degraded, they will think that environmental pollution threatens their health and reduces their quality of life, and then take actions to reduce pollution [46]. Similarly, when people have a good experience of the surrounding environment quality, their psychological comfort will be improved, and their evaluation of the government’s environmental governance behavior will also rise [47]. Therefore, the perception of environmental pollution not only affects the individual’s environmental protection behavior, but also affects the individual’s evaluation of the government’s environmental governance behavior.

According to the analytical logic of the environmentally driven hypothesis, it can be found that people’s experience of surrounding environmental quality stimulates their environmental protection behavior, and they also evaluate the government’s environmental protection behavior according to the level of surrounding environmental quality [48]. In the process of environmental perception, individuals generate their cognition, attitude, and emotion, which serve as the psychological basis for themselves, and transfer this attitude and emotion to judge the government’s satisfaction with environmental governance [49]. People’s dissatisfaction with the surrounding environment will be attributed to the government’s inadequate work and dissatisfaction with the government’s environmental governance. The characteristics of the surrounding objective environment are transformed into individual subjective psychological characteristics [50]. If individuals are often exposed to air pollution, or cannot get clean drinking water, or are often exposed to dust, or inhale harmful or toxic gases, then they are unlikely to have a high degree of satisfaction with environmental governance, and more likely to complain and be dissatisfied with the government [51]. For the government, it is necessary to face the public’s perception of environmental pollution, which becomes an important content to evaluate the government governance performance. Based on this, the following hypothesis is proposed:

**Hypothesis** **2** **(H2):** *There is a positive and significant relationship between environmental pollution perception and environmental governance satisfaction.*

## 3. Materials and Methods

### 3.1. Data Sources

The data used in this study come from the Chinese Social Survey (CSS). The survey was launched in 2005 by the Institute of Sociology of the Chinese Academy of Social Sciences as a large-scale continuous sample survey nationwide. To ensure the scientific nature and reliability of the survey data, the CSS survey ensures the scientific rigor of the survey from multiple aspects. In the sampling section, the CSS survey uses the national census data to design the sampling box; in the management link, the CSS survey, relying on universities and scientific research institutions across the country, established a local investigation team, set up 3–5 days of supervision, visitor training courses, and a variety of visit simulation training, and the research team developed the “field group work method”; in the quality control link, a certain proportion of questionnaires will be rechecked at each survey point, provincial level, and national level to ensure the quality of questionnaires, and all questionnaires will be input twice. The sampled respondents covered more than 150 districts and counties, and more than 600 villages/neighborhood committees across the country. The survey data of CSS has been widely used in China and has been recognized by some authoritative experts in the field. Some scholars have published corresponding papers internationally using the data, such as Wang [52], Li [53], Wei [54], etc. 

This study used the survey data of CSS2019, covering 31 provinces/autonomous regions/municipalities in China, and a total of 10,283 survey samples were selected. The contents of the survey include basic family information, personal work situation, family economic situation, living conditions, environmental pollution problems, social security, social trust and social justice, social values, and social evaluation, etc. According to the needs of this study, the seriously missing samples were eliminated, variables related to government trust, environmental pollution perception, and environmental governance satisfaction were selected, and extreme values and outliers of each variable were deleted. Finally, 3872 samples were screened out.

The characteristics of the 3872 samples are as follows, as shown in Table 1: in terms of gender, female respondents account for 54.78% and male respondents account for 45.22%; in terms of ethnicity, the proportion of Han respondents was 91.17%, and the proportion of minority respondents was 8.83%. In terms of household registration, the urban and rural respondents accounted for 56.90% and 43.10%, respectively; in terms of age, the respondents aged 50–59 were the most, accounting for 26.83%, followed by the respondents aged 60 and above, accounting for 24.85%. In terms of education level, the respondents with junior high school education level are the most, accounting for 32.46%, followed by the respondents with primary school education level, accounting for 21.28%, and the respondents with senior high school education level, accounting for 19.58%. In terms of marital status, married respondents accounted for 80.84% and unmarried respondents accounted for 12.22%. In terms of political status, 80.22% of the respondents were from the masses, and 11.39% were from Communist Party of China members. In general, the interviewed samples are in line with the objective reality and can be statistically analyzed.

### 3.2. Measurements

#### 3.2.1. Satisfaction with Environmental Governance

The dependent variable in this paper is environmental governance satisfaction, and the 2019 CSS questionnaire examines subjective satisfaction. As shown in Table 2, the topic is set as “How are you satisfied with the current government’s environmental governance”, and the answer is set as four classification variables, which are very poor, not so good, better, and very good, respectively, and the values are assigned from 1–4. The CSS questionnaire does not adopt the five-level Likert scale here, because the answer of “general” is excluded from the four classification variables, which can reduce the fuzziness and uncertainty of respondents’ answers [55].

#### 3.2.2. Government Trust

Government trust is a basic social and political relationship between the public and the government. Government trust includes the public’s reasonable expectation of government and the government’s response to public expectation [56]. At present, there are three ways to measure the trust of government in academia. The first way is to measure the trust of different government agencies, including government courts, public security, environmental protection, civil affairs, and other departments. The second measure measures different levels of government, including central government, provincial government, municipal government, county government, and township government. The third measure is the measurement of government workers, including workers at different levels and departments. The 2019 CSS questionnaire takes the second measuring way, measuring the trust of central government, district and county governments, and township and township governments. The answer is set as four classification variables, which are total distrust, less trust, trust, and full confidence, respectively, and assigned values from 1–4.

#### 3.2.3. Environmental Pollution Perception

Environmental pollution perception is the perception of the surrounding environment, indicating the subjective psychological response of the object. According to the Bulletin on the State of China’s Ecology and Environment, air and water pollution are serious pollution problems at present. The government proposes to win the battle of blue sky and clear water [57]. Noise pollution is physical pollution, which is harmless to people. Only when the dose in the environment is too high, it will cause pollution or abnormality. Road traffic noise, rail traffic noise, shop and restaurant noise, and housing decoration noise will all have an impact on people’s lives [58]. Therefore, the 2019 CSS questionnaire measures the perception of environmental pollution from three aspects, namely air pollution, water pollution, and noise pollution. The three are the relatively serious environmental pollution problems faced by the public at present. The designed topic is “Are the following phenomena serious in the area where you currently live?” The range of environmental experience is around the living community, and the answers are very serious, more serious, not too serious, and no such phenomenon, and the values are assigned from 1 to 4. The higher the score of the respondents, the better the Environmental perception.

#### 3.2.4. Control Variables

Reference to the above research results, this paper also considers some control variables, which are gender, age, education level, personal income level, household income level, socioeconomic status, life ideal degree, life happiness degree, and social tolerance degree. Most of these variables have been mentioned in the literature review part, specific settings are shown in Table 2.

### 3.3. Analytical Methods

In this study, the explained variable is environmental governance satisfaction, which is a four-category ordered variable, which is more suitable for the ordered logistic regression model. However, due to the fact that government trust, environmental pollution perception, and control variables are mostly four-categorical and five-categorical variables, the output independent variables of the ordered logistic regression model are relatively miscellaneous, which is not convenient to the present and interpreting the results. To facilitate the interpretation of the regression results and to take into account the comparison of the importance of subjective factors to the public, the optimal scale regression model using SPSS24.0 (IBM, Almond, NY, USA) software is selected. The model’s basic idea is to analyze the strength of the variable types of influence on the dependent variable, in the guarantee under the premise of the relationship between each variable of linear, through certain methods for repeated iterations, as the original classification variables to find an optimal quantitative score, with this rating instead of the original variables for subsequent analysis, and the best regression equation fitting [59]. The advantage of this model is that it can rank the influence importance of independent variables and reflect the size of the influence effect of independent variables.

The optimal scale regression model is a statistical regression model for regression analysis of ordered and unordered multi-categorical variables. The basic model is as follows:

$$Y=\sum_{i=1}^{n}\beta_{i}\chi_{i}+\varepsilon$$

Taking the regression of government trust and satisfaction with environmental governance as an example, *Y* is the public satisfaction with environmental governance after standardization, *χ_i_* is government trust, *n* is the number of independent variables, *β_i_* is the standardized regression coefficient of independent variables, and *ε* is the random error term of regression.

## 4. Results

### 4.1. Describe Statistical Analysis

Of the 3872 respondents, 2024 respondents, accounting for 52.27%, said they were satisfied with environmental governance. A total of 897 respondents, accounting for 23.17%, answered very well; 779 respondents, accounting for 20.12%, thought environmental governance was not very good, while the remaining 172 respondents, accounting for 4.44%, thought it was very bad. Adding for the people answering better and very good, the number of respondents reached 2921, accounting for 75.44%. Overall, the public’s satisfaction with environmental governance is relatively high.

**Figure 1:**
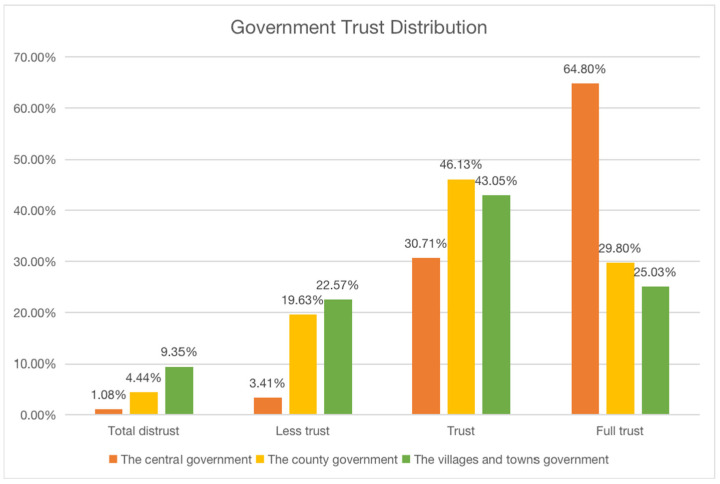
Descriptive analysis of government trust level.

Figure 1 shows the distribution of trust levels in government. The central government accounted for 64.80% on full trust level, accounting for the highest proportion, district and county governments accounted for 29.80% on full trust level, and town and township governments accounted for 25.03% on full trust level. It can be seen that the proportion of trust is decreasing from central to township. On a comparative trust level, the trust level of district and county governments is 46.13%, township governments is 43.05%, and the central government is 30.71%, among which, district and county governments are higher. Adding that for the respondents who answered “Trust” to the respondents who answered “Full trust”, the trust level of the central government, district and county governments, and township governments is 95.51%, 75.93%, and 68.08%, respectively. The trust level of the central government is much higher than that of the local government, showing a distribution pattern of “strong central government and weak local government”, which is manifested as “the differential government trust” [60].

Table 3 presents the frequency analysis of environmental pollution perception. In terms of air pollution, the proportion of very serious, more serious, not too serious, and no such phenomenon was 10.90%, 14.05%, 46.51%, and 28.54%, respectively. Most respondents thought air pollution was not serious. In terms of water pollution, 41.14% of the respondents thought it was not too serious, and 30.81% thought it was no such phenomenon. In terms of noise pollution, the proportion of those who were not too serious and those who thought here was no such phenomenon, which was 38.77% and 38.53% respectively. In general, the public’s perception of air pollution, water pollution, and noise pollution was relatively good, which depended on the government’s efforts to promote environmental governance in recent years and embed the concept of green ecological protection into economic development.

### 4.2. Regression Analysis of Environmental Governance Satisfaction

Table 4 presents the regression model of the impact of government trust and environmental pollution perception on environmental governance satisfaction. In this study, a stepwise regression method was adopted. Control variables were incorporated into the model to obtain Model 1, three variables of environmental pollution perception were incorporated into the regression model to obtain Model 2, and three variables of government trust were incorporated into the model to obtain Model 3. The tolerance values of all independent variables in the three models were greater than 0.1 before and after transformation, indicating that there was no multicollinearity problem of independent variables. The F values of the three regression models were 15.881, 21.185, and 24.881, respectively, and the ANOVA results of the three models were all less than 0.000, which met the significance requirement of 0.05, indicating that the model fitting results were valid. The R square of the model increased from 0.114 to 0.242 and from 0.106 to 0.232 after adjustment, indicating that with the addition of environmental pollution perception and government trust, the fitting effect of the model is gradually improving. In general, the model results can be analyzed.

In Model 1, gender does not have a significant impact, there is no difference between men and women in environmental governance satisfaction, personal income level does not have a significant impact, but household income level has a significant impact, and other control variables have a significant impact. In Model 2, the gender still does not have a significant effect, personal income level has a significant effect, which illustrates the influence effect of personal income level is unstable, and age, education level, household income level, socioeconomic status, life ideal degree, life happiness degree, and social tolerance degree have significant effect of the degree of social tolerance. The control variables of the specific analysis are as follows:

Age has a significant effect. With the increase in age, people’s satisfaction with environmental governance is higher. The possible explanation is that the older people are, the more they care about environmental governance issues. With the accumulation of age, they can experience the improvement of the quality of the environment around them and enjoy the environmental governance products of the government. Education level has a significant impact. With the improvement of education level, the satisfaction with environmental governance will also increase. The possible explanation is that people with a higher education level have more opportunities to participate in environmental governance, stronger subjective efficacy, and higher environmental tolerance. Both household income level and socioeconomic status have significant positive effects on the satisfaction of environmental governance. The possible explanation is that households with a higher income level have higher requirements for green living and can purchase housing in green environmental protection communities with a sounder infrastructure, and the environmental sanitation treatment in their living areas is more efficient and timely. They can also enjoy more quality services by purchasing environmental protection services. Both life ideal degree and life happiness degree have significant influence on the satisfaction degree of environmental governance. On the one hand, environmental governance is an important factor that constitutes life ideal and life happiness. Good environmental governance can improve personal happiness level. On the other hand, people with a high sense of life happiness have a positive and optimistic attitude and an upward value concept, which is transferred to the evaluation of environmental governance satisfaction. Between social tolerance level and environmental improvement, satisfaction has a significant effect. In addition, the higher the degree of social tolerance is, the less tension there is between the government and the people, and people’s understanding of government behavior will be more forgiving, even if in the government there exist certain problems on environmental governance, or did not achieve the desired effect, and they also believe that the government has tried our best.

In Model 3, all three variables of environmental pollution perception have a significant impact on environmental governance satisfaction. With the improvement of environmental pollution perception preference, people’s satisfaction with environmental governance will also increase, proving hypothesis H2. The effects of air pollution, water pollution, and noise pollution on environmental governance satisfaction were 0.167, 0.119, and 0.052, respectively, and the effect of air pollution is large. People’s perception of environmental pollution will form subjective emotions, which will have an impact on people’s evaluation. Positive emotions are conducive to the improvement of satisfaction, while negative emotions will reduce satisfaction. Environmental pollution perception can reflect the performance of government work. A good environmental pollution perception indicates that the government has taken positive measures in environmental health management. These environmental protection policies have improved the living environment and provided a good living environment for the people.

In Model 3, the three variables of government trust all have a positive and significant impact on environmental governance satisfaction, proving hypothesis H1. The influence effect of the central government, district and county governments, and township governments on environmental governance satisfaction was 0.025, 0.086, and 0.207, respectively. With the improvement of government trust, people’s evaluation of environmental governance satisfaction was higher. The government trust of the public comes from past experience and facts. High government trust establishes the image of the government in the people’s mind, establishes the authority, and obtains the legitimacy. People’s trust in the government is transformed into expectations for the government’s future governance. In order to maintain its image and authority, the government will try its best to cater to the needs of the people and meet their interests. Under the background of high government trust, the government will reduce selective enforcement, distorted enforcement, discounted enforcement, non-enforcement, and other behaviors, and actively take measures to improve the quality of people’s living environment, provide high-quality environmental public goods, and improve government governance performance [61].

## 5. Discussion

In order to explore the impact of government trust and environmental pollution perception on people’s satisfaction with environmental governance, this paper uses the optimal scale regression model as an analytic tool to explore the relationship between the three factors. The results of the stepwise regression model show that government trust and environmental pollution perception have a positive and significant impact on people’s satisfaction with environmental governance, which proves the hypotheses H1 and H2 proposed in this paper. Both government trust and environmental pollution perception are subjective psychological values generated objectively by the public, and then the satisfaction of environmental governance is evaluated. Government trust is based on the impact of government performance and public expectation on environmental governance satisfaction. The government needs to respond to public demands with public goods and government performance to further provide legitimacy for government governance. Environmental pollution perception is the objective feeling of the government’s pollution control, which directly comes from the people’s life practice. It means whether the implementation of the government’s environmental protection policy can improve the environmental quality of the people, and thus improve the satisfaction of environmental governance.

In the perception of environmental pollution, the effect of air pollution is 0.167, which is larger than that of water pollution and noise pollution. This is the same as the findings of Zhang et al., who believed that air pollution has a more important impact on public environmental satisfaction than meteorological factors [62]. The study of Pu showed that 76% of respondents were very concerned about air pollution and were worried about the harmful consequences of exposure to air pollution and were more adamant in their attitude [63]. Compared with water pollution and noise pollution, soot, dust, sulfur oxides, nitrogen oxides, carbon compounds, and sulfur dioxide, etc., in the air directly threaten respiratory function and lung function after being breathed by the human body, causing harm to human life. Long-term exposure to air pollution will seriously reduce people’s subjective well-being [64]. The perception of environmental pollution may be more obvious than socialization and cultural factors in influencing people’s attitudes and reactions to the environment [65].

Therefore, people are more sensitive to air pollution. At the same time, from the point of view of the government’s environmental governance, waste gas pollution treatment is easier than water pollution treatment. Waste gas treatment only needs to control the emission of waste gas companies and enterprises to directly reduce the emissions of waste gas, but water pollution control requires the main emitter to install a variety of purification machines, to achieve standard emissions. The government can invest less energy in air pollution control and get obvious expected benefits in the short term, while water pollution control needs a lot of energy in the early stage and the return of its benefits is very slow [66]. Air pollution control is also in line with the government’s environmental protection policy and governance performance needs. However, in the long run, all kinds of environmental pollution should be included in the scope of control to reduce the harm to human health.

In the aspect of government trust, the distribution of the government trust level does not match the distribution of its influence effect. The level of government trust is on the rise from township, district, and county to the central government. The higher the trend of government trust is, the higher the level of government trust is, which shows a pyramid shape distribution. However, the influence of government trust on satisfaction with environmental governance shows an opposite trend, and the influence from the central government, district, county, and township shows an upward trend, showing an inverted pyramid structure. It can be seen that the level of government trust does not coincide with the effect of government trust. The trust level of the Chinese government is in the state of “differential government trust” [67], which is related to Chinese Confucian history and culture, and culture shapes the distribution of government trust level [68]. The level of government trust in China is not the same as in the United States. In the United States, the distribution of government trust among different levels of government presents an inverted pyramid structure, that is, people’s trust in the federal government is slightly lower than that of the state government, which is slightly lower than that of the local government [69]. For this reason, Fredrickson proposed the “paradox of distance” to explain the distribution of government trust in the United States. The closer the distance is, the more the public thinks the officials are hardworking, competent, and active, while the farther the distance is, the more the officials are lazy, incompetent, and passive [70]. As a result, the public has less trust in the high-level government in the United States, and more trust in the city and state governments.

According to the normal logic, the higher the level of government trust, its impact on environmental governance satisfaction should be greater. However, this article does not confirm this logic, but the opposite. Township governments have a greater impact on environmental governance satisfaction. This article explains from the angle of political contact and political interaction. From the perspective of the general public, it is precisely because of the close distance between township governments and individuals that individuals can interact with township government officials, express their interests, and share public goods from the environmental protection policies of grassroots governments [71]. At the same time, just because of the close distance between the people and the township government, the people can realize the effective interaction with the township government, and increase the understanding of the government. More importantly, the implementation of environmental protection policies is carried out by the grassroots government and its staff. Township governments need to realize the integration of various resources to promote the implementation of environmental governance policies [72]. People living in the jurisdiction area can directly observe the environmental governance behavior and interaction of township grassroots government staff. In addition, it should be noted that the greater impact of township governments on environmental governance satisfaction does not mean that district and county governments and the central government play a lower role in influencing environmental governance satisfaction [73]. If there is no environmental protection policy formulated by the central government, there is no basis for township governments to implement environmental protection policies.

## 6. Limitation

There are four limitations in this study: Firstly, the study used data from a 2019 survey by the Chinese Academy of Social Sciences. Researchers do not have a detailed understanding of the data sampling process, and the research can only be conducted based on existing questionnaires. For example, the answers of independent variables and dependent variables are mostly set as four-categorical variables, rather than five-level Likert scales. The research theory is not well supported, which limits the further exploration of this study; secondly, public satisfaction with environmental governance is the result of comprehensive factors. This paper mainly investigates the influence of government trust and environmental pollution perception on environmental governance satisfaction, and other influencing factors have not been investigated. Thirdly, the relationship between government trust and environmental pollution perception has not been explored, and whether government trust has a mediating effect between environmental pollution perception and environmental governance satisfaction needs to be further explored. Finally, the data in this study are from the survey in China, and whether it is universal in other countries needs to be further verified.

## 7. Conclusions

Based on the survey data of CSS2019, 3872 survey samples were analyzed, and the optimal scale regression model was used to explore the impact of government trust and environmental pollution perception on environmental governance satisfaction. The results show that the public’s satisfaction with environmental governance is relatively high on the whole, and both government trust and environmental pollution perception have a significant impact on environmental governance satisfaction. Hypothesis H1 and H2 are confirmed. Government trust plays a role through “expectation-response”, and environmental pollution perception plays a role through subjective feeling evaluation. It is found that people’s trust level in government gradually rises from township, district, and county to the central government in a pyramid structure, which is different from the distribution of government trust level in the United States. The influence of government trust on the satisfaction of environmental governance shows an inverted pyramid structure, and the township government has the largest effect, which does not match the distribution of the government trust level. The relatively close distance between the people and the township government increases the opportunity to contact the person in charge and the staff of the township government. The people can realize the interaction with the township government and express their interest needs in the interaction. The public can observe the environmental governance measures actively taken by the township government staff, share the public goods of environmental governance, improve their sense of gain, and increase their satisfaction.

First, the government should strengthen ecological construction and environmental protection, and put environmental protection in a prominent position. The central government should further strengthen the control of air pollution, increase the investment of funds for air pollution control, formulate air quality plans and supervision plans for air pollution sources, and incorporate air pollution quality assessment into the promotion index system for local officials. Second, promoting “government transparency” and “government response”, disclosing information related to environmental governance to the public, increasing channels for communication and interaction between the public and the government, responding to the needs of the public, establishing a good image of the government, and thus improving the public’s trust in the government. Third, it is time to improve the environmental governance system of grassroots governments, build a diversified environmental governance model of “government, enterprises and people”, and improve the environmental governance ability of township governments by reconstructing their administrative processes, optimizing government services, and promoting interactions between government and people, so that township governments can play a greater role in environmental governance.

## Figures and Tables

**Table 1:** Characteristics of the survey sample.

Characteristics of the Indicators	Classification	Frequency	The Proportion (%)	The Standard Deviation
Gender	Female	2121	54.78	0.50
	Male	1751	45.22	
Nationality	The Han nationality	3530	91.17	0.28
	Minority	342	8.83	
Household registration	Urban	2203	56.90	0.50
	Rural area	1669	43.10	
Age	Under the age of 30	469	12.11	1.33
	30–39	630	16.27	
	40–49	772	19.94	
	50–59	1039	26.83	
	60 and above	962	24.85	
Education level	Illiteracy	283	7.31	1.19
	Primary school	824	21.28	
	Junior high school	1257	32.46	
	High school	758	19.58	
	Junior college or above	750	19.37	
Marital status	Unmarried	473	12.22	0.56
	Married	3130	80.84	
	Divorced	116	3.00	
	Widowed	153	3.95	
Politics status	Member of Communist Party of China	441	11.39	1.05
	Member of communist youth league of China	314	8.11	
	The democratic parties	11	0.28	
	The masses	3106	80.22	
In total		3872	100	

**Table 2:** Variable definitions and assignments.

Variable Type	Variable Name	Variable Definition	Mean Value	Variable Definition
The dependent variable	Environmental governance satisfaction	Very poor = 1; Not so good = 2; Better = 3; Very good = 4	2.94	0.78
Control variable	Gender	Female = 1; Male = 2	1.45	0.50
	Age	Under 30 = 1; 30–39 = 2; 40–49 = 3; 50 to 59 = 4; 60 and above = 5	3.36	1.33
	Education level	Illiteracy = 1; Primary school = 2; Junior high school = 3; High school = 4; Junior College or above = 5	3.22	1.19
	Personal income level	Low income = 1; Low and middle income = 2; Middle income = 3; Middle and high income = 4; High income = 5	2.69	1.55
	Household income level		3.07	1.55
	Socioeconomic status	Low status = 1; The middle and lower = 2; The middle = 3; Above middle = 4; High status = 5	2.40	0.92
	Life ideal degree	Strongly disagree = 1; Disagree = 2; Comparative agreement = 3; Strongly agree = 4	2.80	0.86
	Life happiness degree		3.18	0.79
	Social tolerance degree	Very intolerant = 1; Less tolerant = 2; General = 3; More tolerant = 4; Very tolerant = 5	3.68	0.90
The government trust	The central government	Total distrust = 1; Less trust = 2; Trust = 3; Full confidence = 4	3.59	0.61
	County level government		3.01	0.82
	Township government		2.84	0.91
Environmental pollution perception	Air pollution	Very serious = 1; More serious = 2; Not too serious = 3; No such phenomenon = 4	2.93	0.93
	Water pollution		2.91	0.96
	Noise pollution		3.07	0.94

**Table 3:** Description and analysis of environmental pollution perception (Unit: Pcs, %).

Air Pollution	Frequency	Proportion	Water Pollution	Frequency	Proportion	Noise Pollution	Frequency	Proportion
Very serious	422	10.90	Very serious	451	11.65	Very serious	348	8.99
More serious	544	14.05	More serious	635	16.40	More serious	531	13.71
Not too serious	1801	46.51	Not too serious	1593	41.14	Not too serious	1501	38.77
There is no such phenomenon	1105	28.54	There is no such phenomenon	1193	30.81	There is no such phenomenon	1492	38.53
Sample: 3,872,100								

**Table 4:** Regression results of environmental governance satisfaction.

Variable	Model 1		Model 2		Model 3	
	β	Standard Error	β	Standard Error	β	Standard Error
Control variable						
Gender	0.017	0.013	0.017	0.013	0.010	0.011
Age	0.056 ***	0.016	0.078 ***	0.017	0.071 ***	0.016
Education level	0.097 ***	0.020	0.045 ***	0.016	0.047 ***	0.015
Personal income level	0.017	0.013	0.027 *	0.014	0.029 ***	0.014
Household income level	0.075 ***	0.017	0.059 ***	0.018	0.050 ***	0.016
Socioeconomic status	0.070 ***	0.017	0.065 ***	0.016	0.055 ***	0.016
Life ideal degree	0.100 ***	0.020	0.074 ***	0.020	0.045 ***	0.016
Life happiness degree	0.100 ***	0.019	0.074 ***	0.018	0.059 ***	0.016
Social tolerance degree	0.155 ***	0.018	0.109 ***	0.018	0.064 ***	0.016
Environmental pollution perception						
Air pollution			0.185 ***	0.027	0.167 ***	0.024
Water pollution			0.138 ***	0.021	0.119 ***	0.021
Noise pollution			0.060 ***	0.018	0.052 ***	0.018
The government trust						
The central government					0.025 *	0.015
County-level government					0.086 ***	0.028
Township government					0.207 ***	0.029
F	15.881		21.185		24.881	
Sig.	0.000		0.000		0.000	
R squared	0.114		0.181		0.242	
Adjusted R square	0.106		0.173		0.232	
Sample	3872		3872		3872	

Note: 1. * *p* ≤ 0.05, *** *p* ≤ 0.001; 2. Limited by the length of the table, the tolerance values of the independent variables of the three models before and after transformation are not presented.

## Data Availability

The data are available in a publicly accessible repository: http://css.cssn.cn/css_sy/ (accessed on 30 March 2022).
